# Machine learning for early detection of sepsis: an internal and temporal validation study

**DOI:** 10.1093/jamiaopen/ooaa006

**Published:** 2020-04-11

**Authors:** Armando D Bedoya, Joseph Futoma, Meredith E Clement, Kristin Corey, Nathan Brajer, Anthony Lin, Morgan G Simons, Michael Gao, Marshall Nichols, Suresh Balu, Katherine Heller, Mark Sendak, Cara O’Brien

**Affiliations:** o1 Department of Medicine, Division of Pulmonary, Allergy, and Critical Care Medicine, Duke University, Durham, North Carolina, USA; o2 Department of Statistics, Duke University, Durham, North Carolina, USA; o3 John A. Paulson School of Engineering and Applied Sciences, Harvard University, Cambridge, Massachusetts, USA; o4 Department of Medicine, Division of Infectious Diseases, Duke University, Durham, North Carolina, USA; o5 Duke Institute for Health Innovation, Durham, North Carolina, USA; o6 Duke University School of Medicine, Durham, North Carolina, USA; o7 Department of Medicine, Durham, North Carolina, USA

**Keywords:** adult, sepsis/mortality, electronic health records/statistics and numerical data, machine learning, decision, support systems, clinical, emergency service, hospital/statistics and numerical data, hospitalization/statistics and numerical data, ROC curve, retrospective studies

## Abstract

**Objective:**

Determine if deep learning detects sepsis earlier and more accurately than other models. To evaluate model performance using implementation-oriented metrics that simulate clinical practice.

**Materials and Methods:**

We trained internally and temporally validated a deep learning model (multi-output Gaussian process and recurrent neural network [MGP–RNN]) to detect sepsis using encounters from adult hospitalized patients at a large tertiary academic center. Sepsis was defined as the presence of 2 or more systemic inflammatory response syndrome (SIRS) criteria, a blood culture order, and at least one element of end-organ failure. The training dataset included demographics, comorbidities, vital signs, medication administrations, and labs from October 1, 2014 to December 1, 2015, while the temporal validation dataset was from March 1, 2018 to August 31, 2018. Comparisons were made to 3 machine learning methods, random forest (RF), Cox regression (CR), and penalized logistic regression (PLR), and 3 clinical scores used to detect sepsis, SIRS, quick Sequential Organ Failure Assessment (qSOFA), and National Early Warning Score (NEWS). Traditional discrimination statistics such as the C-statistic as well as metrics aligned with operational implementation were assessed.

**Results:**

The training set and internal validation included 42 979 encounters, while the temporal validation set included 39 786 encounters. The C-statistic for predicting sepsis within 4 h of onset was 0.88 for the MGP–RNN compared to 0.836 for RF, 0.849 for CR, 0.822 for PLR, 0.756 for SIRS, 0.619 for NEWS, and 0.481 for qSOFA. MGP–RNN detected sepsis a median of 5 h in advance. Temporal validation assessment continued to show the MGP–RNN outperform all 7 clinical risk score and machine learning comparisons.

**Conclusions:**

We developed and validated a novel deep learning model to detect sepsis. Using our data elements and feature set, our modeling approach outperformed other machine learning methods and clinical scores.

## INTRODUCTION

Mortality rates in patients with untreated sepsis can exceed 30%.^1^^,^^2^ As a leading cause of mortality,^3^ sepsis represents a significant burden to the patient, clinician, and healthcare system. Protocol-driven care bundles improve clinical outcomes,^4^^,^^5^ but require early detection of sepsis, which remains elusive even for experienced clinicians.

In 2016, a new consensus definition (Sepsis-3) was published, which utilizes the Sequential Organ Failure Assessment (SOFA) and a newly developed quick Sequential Organ Failure Assessment (qSOFA) to identify patients at risk for poor outcomes due to sepsis.^6^ The Sepsis-3 criteria have been criticized for detecting sepsis late in the clinical course.^4^^,^^7^^,^^8^ The Centers for Medicare and Medicaid Services (CMS) continue to use an older sepsis definition based on the presence of the systemic inflammatory response syndrome (SIRS) for the purposes of measuring compliance with the sepsis quality of care bundles (SEP-1 measure).^4^^,^^7^

Quality improvement programs implemented at individual health systems have improved outcomes for patients with sepsis.^9^^,^^10^ However, overall compliance with recommended treatment remains poor. Deep learning is a suite of novel machine learning methods that have achieved performance on many challenging tasks.^11^

The present study carries out 3 analyses to better characterize how a deep learning approach can detect sepsis early in the emergency department (ED) and pre-intensive care unit (ICU) inpatient setting. The deep learning model was specifically designed to detect the first episode of sepsis between presentation to the ED and discharge home, inpatient mortality, or transfer to an ICU. First, we compare the performance of our previously derived deep learning approach^12^^,^^13^ to clinical scores that are commonly used to identify patients at risk of sepsis. Second, we compare the performance of our model to previously published machine learning methods used to predict sepsis. Third, we test how well our model, clinical scores, and previously published machine learning methods generalize to a planned future implementation.

## MATERIALS AND METHODS

### Datasets

This retrospective, single-center study analyzed electronic health record (EHR) data from a quaternary academic hospital with 43 000 inpatient and 1 million outpatient visits annually. This study is reported as per the Transparent Reporting of a Multivariable Prediction Model for Individual Prognosis or Diagnosis (TRIPOD) guidelines^14^ and was approved by the Duke University Health System Institutional Review Board (Pro00093721, Pro00080914).

The model development cohort consisted of all inpatient admissions that began in the ED between October 1, 2014 and December 1, 2015. Patients under the age of 18 were excluded. Hospital admissions that did not originate in the ED (eg, direct admission; scheduled surgery) and ED encounters that did not result in inpatient admission were also excluded from the model development cohort. Patients who developed sepsis within 1 h of presentation to the ED were excluded. Encounter data began at presentation to the ED. Encounters that did not result in sepsis ended at time of discharge, time of death, or time of ICU transfer. Encounters that did result in sepsis ended at time of the first sepsis episode. All data after discharge, the first sepsis episode, ICU transfer, or death were excluded from model development. Patients who developed sepsis after transfer to an ICU were included and treated as control cases. Curated features included structured static variables, such as demographic, encounter, and pre-admission comorbidity data, as well as dynamic variables, such as vital sign, medication, and lab data. Vital sign measurements, medication administrations, and lab collections that occurred between the encounter start and end times were included.

There is no gold standard for the definition of sepsis. Various definitions of sepsis have been described in the literature which partition out specific populations to meet study or epidemiological needs. Sepsis was defined in our data by the presence of 2 or more SIRS criteria, a blood culture order, and at least one element of end-organ failure (Supplementary Table S1). Our definition was based upon prior efforts by our study team.^15^ A similar sepsis definition has been used for model development efforts at peer institutions that developed at least 2 other published models, and this definition aligns with the CMS definition.^16^^,^^17^

We compared our sepsis definition with Sepsis 1, Sepsis 3, and the Centers for Disease Control (CDC) Adult Sepsis Event.^17^ The Sepsis-1 and Sepsis-3 definitions were computed using SIRS and qSOFA criteria. An order for any culture served as a proxy for clinician suspicion for infection to enable the Sepsis-1 and Sepsis-3 definition to be automatically computed from the EHR without manual chart review. The CDC Adult Sepsis Event surveillance definition is based on the Sepsis-3 framework of suspected infection with organ dysfunction.^18^ Sensitivity, specificity, positive predictive value (PPV), and negative predictive value were calculated for each definition using CDC Adult Sepsis Events as the gold standard.

A separate temporal validation cohort was curated from the same site. The cohort was not limited to inpatient admissions but included all ED encounters between March 1, 2018 and August 31, 2018. The same variables, inclusion and exclusion criteria, and outcome definition were applied. Unlike the model development cohort, the temporal validation cohort included encounters that began in the ED that did not result in inpatient admission.

A total of 86 variables were automatically curated^19^ for each cohort, including patient demographics, comorbidities, vital signs, medication administrations, and labs (Supplementary Table S2). In total, the model development cohort contained over 32 million data points.

### Model development

We built on prior work coupling multi-output Gaussian processes (MGPs) and recurrent neural networks (RNNs) (hereafter called MGP–RNN).^12^^,^^13^ RNNs are a form of deep learning designed to ingest time series data and handle sequences of variable length.^20^ A core feature of any deep learning method is the ability to capture complex relationships between input variables. RNNs can use a patient’s complete pre-encounter and encounter data to predict an outcome while maintaining temporal relationships.^21–23^ RNNs generally require evenly spaced inputs, even if the overall lengths of encounters differ. A variety of imputation strategies have been used to model inputs that are irregularly sampled and often missing in EHR data,^24–26^ including multitask learning, which models relationships between time series.^27^ An MGPs are a type of multitask learning that is probabilistic and maintains uncertainty about the true value.

Dynamic features (eg, vitals; labs) are sampled every hour from the MGP along with missingness indicator variables and fed into the RNN. Static features are replicated every hour and fed into the RNN. No minimum amount of data is required to generate a risk score. At each timepoint *t*, the likelihood of sepsis is computed and evaluated against whether or not the patient develops sepsis between time *t* and *t* plus 4 h.

The model development cohort was divided into training, test, and internal validation subsets. The training subset contained 80% of all encounters. The remaining encounters were evenly split between a test subset for hyperparameter selection and an internal validation subset. The internal validation subset was blinded to all methods until final evaluation. Each model was trained on the training subset until time of sepsis. For control encounters, data until a randomly chosen timepoint mid-encounter was used. Every model generated a risk score each hour starting 1 h after admission.

The performance of MGP–RNN was assessed using 2 sets of comparisons . First, we compared the performance of the MGP–RNN to SIRS,^28^ National Early Warning Score (NEWS),^29^ and qSOFA.^6^ Next, we compared the performance of the MGP–RNN to a Lasso-penalized Cox regression (CR),^30^ random forest (RF),^31^ and penalized logistic regression.^32^ Both sets of comparisons assess global performance of methods as well as performance as time passes following presentation to the ED.^25^ This analysis demonstrates the ability of the various approaches to detect sepsis as early in the hospital course as possible.

### Temporal validation

Finally, we compared the performance of MGP–RNN against all 7 clinical scores and machine learning methods on a temporal validation cohort. The temporal validation cohort represents a planned future implementation in an adult ED.

### Statistical analysis

Evaluation metrics included area under the receiver operating characteristic curve (AUC). Lastly, we fix the number of alerts allowed per hour and report the number of sepsis cases identified early per day. This reflects the workflow constraint of needing to limit the number of alerts fired to front-line clinicians. Model performance is calculated on the 10% internal validation subset and on the temporal validation cohort.

Models generate risk scores every hour and we calculate performance using 2 approaches. To assess global performance, similar to prior work,^13,^^26^^,^^33^^,^^34^ metrics are calculated using the maximum score within independent 12-h windows. True positives are high-risk scores during 12-h blocks immediately preceding a sepsis event. False positives are high-risk scores during 12-h blocks not immediately preceding a sepsis event. To assess performance as time passes following presentation to the ED, metrics are calculated using the maximum score within windows ranging in size from 1-h to 12 h. True positives are high-risk scores during a window followed by a sepsis event within 4 h. False positives are high-risk scores during a window not followed by a sepsis event within 4 h. All model evaluations are completed without an alert ‘snooze’, a time period during which risk scores are suppressed and not considered.

All methods were implemented using the numpy (version 1.14.0), scikit-learn (version 0.18.1), and TensorFlow (version 1.6.0) python packages.

## RESULTS

In the model development cohort, there were 42 979 admissions and sepsis developed in 8160 (19.0%) admissions. In the temporal validation cohort, there were 39 786 encounters and sepsis developed in 2562 (6.4%) encounters. Table 1 presents demographic and clinical characteristics of the model development, internal validation, and temporal validation cohorts. Sepsis was observed early in the hospital course. In the model development cohorts, 3100 (38%) sepsis cases occurred between presentation to the ED and inpatient admission. Furthermore, in the model development cohorts, 791 (9.7% overall; 25.5% of those in the ED) sepsis cases occurred within 1 h of presentation to the ED, and 372 (4.6%) sepsis cases occurred within 1 h of inpatient admission. Supplementary Figure S1 shows the full distribution of time of sepsis within both the model development and temporal validation cohorts. Supplementary Table S3 illustrates the performance of our sepsis definition, Sepsis-1, and Sepsis 3 in detecting CDC Adult Sepsis Events. Notably, our sepsis definition had the highest PPV for identifying patient that ultimately received 4 days of antibiotics to meet the CDC Adult Sepsis Event definition.

**Table 1.** Baseline characteristics of internal development and validation cohorts (90% and 10% of full data), and of temporal validation cohort

Baseline characteristic of cohort	Development, *n* (%) (*N* = 38 682)	Septic development, *n* (%) (*N* = 7347)	Internal validation, *n* (%) (*N* = 4297)	Septic internal validation, *n* (%) (*N* = 813)	Temporal validation, *n* (%) (*N* = 39 786)	Septic temporal validation, *n* (%) (*N* = 2562)
Age (years), mean ± SD	55.9 (18.7)	59.8 (17.1)	56.2 (18.6)	59.6 (17.3)	50.4 (19.5)	59.7 (17.0)
Sex male	18 203 (47.1)	4005 (54.5)	2050 (47.7)	434 (53.4)	18 272 (45.9)	1418 (55.3)
Weight (lbs), mean ± SD	158.8 (19.7)	160.2 (19.8)	158.4 (19.4)	159.7 (19.8)	185.8 (72.1)	184.4 (60.1)
Admission source						
Home/non-healthcare facility	30 063 (77.7)	5072 (69.0)	3363 (78.3)	577 (71.0)	34 848 (87.6)	1854 (72.4)
Transfer from hospital	4930 (12.7)	1498 (20.4)	534 (12.4)	147 (18.1)	2877 (7.2)	538 (21.0)
Missing/other	3689 (9.5)	777 (10.6)	400 (9.3)	89 (10.9)	2061 (5.2)	170 (6.6)
Admission type						
Elective	11 854 (30.6)	571 (7.8)	1338 (31.1)	60 (7.4)	5620 (14.1)	138 (5.4)
Emergency	16 478 (42.6)	4813 (65.5)	1797 (41.8)	522 (64.2)	30 048 (75.5)	1917 (74.8)
Urgent	10 342 (26.7)	1963 (26.7)	1162 (27.0)	231 (27.8)	4118 (10.4)	507 (19.8)
Race						
Black or African American	11 390 (29.4)	2329 (31.7)	1252 (29.1)	255 (31.4)	15 805 (39.7)	931 (36.3)
Caucasian/White	24 317 (62.9)	4661 (63.4)	2681 (62.4)	499 (61.4)	19 701 (49.5)	1454 (56.8)
Missing/other	2975 (7.7)	357 (4.9)	364 (8.5)	59 (7.3)	4280 (10.8)	177 (6.9)
Comorbidities						
Congestive heart failure	5656 (14.6)	1576 (21.5)	627 (14.6)	175 (21.6)	3329 (8.4)	469 (18.3)
Valvular disease	5288 (13.7)	1298 (17.7)	544 (12.7)	137 (16.9)	1975 (5.0)	232 (9.1)
Peripheral vascular disease	4283 (11.1)	1016 (13.8)	474 (11.0)	119 (14.6)	1678 (4.2)	198 (7.7)
Hypertension	18 251 (47.2)	4114 (56.0)	2009 (46.8)	445 (54.7)	11 874 (29.8)	1014 (39.6)
Other neurological disorders	6725 (17.4)	1974 (26.9)	731 (17.0)	226 (27.8)	2538 (6.4)	239 (9.3)
Pulmonary circulation disorders	6917 (17.9)	1830 (24.9)	779 (18.1)	192 (23.6)	4047 (10.2)	338 (13.2)
Diabetes mellitus without chronic complications	6071 (15.7)	1394 (19.0)	685 (15.9)	169 (20.8)	2896 (7.3)	225 (8.8)
Renal failure	6188 (16.0)	1876 (25.5)	673 (15.7)	216 (26.6)	3829 (9.6)	600 (23.4)
Solid tumor without Metastasis	4711 (12.2)	809 (11.0)	525 (12.2)	93 (11.4)	3879 (9.7)	395 (15.4)
Coagulopathy	4503 (11.6)	1588 (21.6)	497 (11.6)	173 (21.3)	1558 (3.9)	341 (13.3)
Obesity	5542 (14.3)	1203 (16.4)	598 (13.9)	133 (16.4)	3213 (8.1)	207 (8.1)
Fluid and electrolyte disorders	10 204 (26.4)	3221 (43.8)	1110 (25.8)	353 (43.4)	4855 (12.2)	668 (26.1)
Anemia	9242 (23.9)	2763 (37.6)	1055 (24.6)	309 (38.0)	3327 (8.4)	396 (15.5)
Depression	6308 (16.3)	1526 (20.8)	715 (16.6)	168 (20.7)	2721 (6.8)	174 (6.8)
Prior sepsis encounters in past year						
0	36 634 (94.7)	6363 (86.6)	4103 (95.5)	727 (89.4)	38 872 (97.7)	2319 (90.5)
1	1514 (3.9)	688 (9.4)	149 (3.5)	64 (7.9)	681 (1.7)	165 (6.4)
2 or more	534 (1.4)	296 (4.0)	45 (1.0)	22 (2.7)	233 (0.6)	78 (3.0)
Overall in-hospital mortality (%)	1257 (3.2)	696 (9.5)	121 (2.8)	59 (7.3)	577 (1.5)	337 (13.2)
Overall length of stay (h), median (25%–75%)	95 (57–168)	167 (95–318)	95 (55–168)	167 (94–315)	13 (5–90)	172 (95–342)
Overall rate of ICU admission (%)	5870 (15.2)	1530 (20.8)	646 (15.0)	166 (20.4)	4598 (11.6)	1148 (44.8)
Septic (%)	7347 (19.0)	7347 (100.0)	813 (18.9)	813 (100.0)	2562 (6.4)	2562 (100.0)

*Note*: For each cohort, characteristics are also broken out among the subgroup of patients who acquire sepsis.

**Figure 1.**
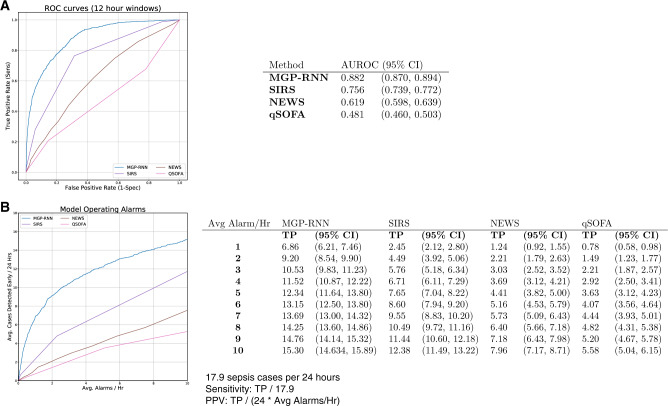
Results of our deep learning model compared with the clinical scores methods. (A) ROC curves for the MGP–RNN and the 3 clinical scores considered, SIRS, NEWS, and qSOFA is shown. The accompanying table lists C-statistics with bootstrap confidence intervals. (B) The average number of sepsis cases each day we expect to detect early before a definition for sepsis is met (ie, a more interpretable version of sensitivity), as a function of how many alarms each method would produce each hour is shown. We limit the average alarms per hour to less than 10, as this is the operating range at which we expect to use in practice. There were an average of 17.9 sepsis cases per 24-h period in the dataset, so sensitivity can be recovered by dividing the reported *y*-axis value in panel B by 17.9. Positive predictive value at a particular threshold can be recovered by dividing the reported *y*-axis value by 24 times the reported *x*-axis value (ie, the average number of alarms per 24-h period). MGP–RNN, multi-output Gaussian process and recurrent neural network; NEWS, national early warning score; QSOFA, quick Sequential Organ Failure Assessment; SIRS, systemic inflammatory response syndrome.

MGP–RNN outperformed SIRS, qSOFA, and NEWS. Figure 1A shows AUC and Figure 1B shows operational metrics fixing the number of alarms per hour. To minimize alarm fatigue, a workflow can be designed that limits the number of alerts prioritized for a clinician to review per hour. Allowing 3 alarms per hour, MGP–RNN captured 10.5 out of 17.9 sepsis cases per day, compared to 5.76 for SIRS, 3.03 for NEWS, and 2.21 for qSOFA.

**Figure 2.**
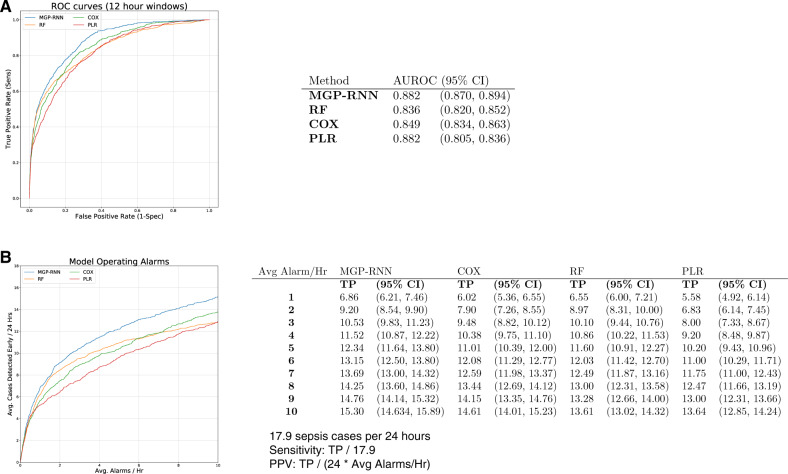
Results of our deep learning model compared with the other machine learning models. (A) ROC curves for the MGP–RNN and the 3 other machine learning models considered, Cox regression, penalized logistic regression, and random forest is shown. The accompanying table lists C-statistics with bootstrap confidence intervals. (B) The average number of sepsis cases each day we expect to detect early before a definition for sepsis is met (ie, a more interpretable version of sensitivity), as a function of how many alarms each method would produce each hour is shown. We limit the average alarms per hour to less than 10, as this is the operating range at which we expect to use in practice. There were an average of 17.9 sepsis cases per 24-h period in the dataset, so sensitivity can be recovered by dividing the reported *y*-axis value in panel B by 17.9. Positive predictive value at a particular threshold can be recovered by dividing the reported *y*-axis value by 24 times the reported *x*-axis value (ie, the average number of alarms per 24-h period). MGP–RNN, multi-output Gaussian process and recurrent neural network; PLR, penalized logistic regression; RF, random forest.

MGP–RNN also outperformed machine learning methods used in previously published sepsis prediction models. Figure 2A shows AUC for each approach and Figure 2B shows operational metrics fixing the number of alarms per hour. Allowing 3 alarms per hour, MGP–RNN captured 10.5 out of 17.9 sepsis cases per day, compared to 9.48 for CR, 8.00 for logistic regression (LR), and 10.10 for RF.

At this threshold yielding an average of 3 alarms per hour, MGP–RNN detects sepsis a median of 5 h in advance (with 25% and 75% quantiles of 2 and 20 h). Supplementary Figure S2 shows the full distribution of how far in advance MGP–RNN detects sepsis in both the internal and temporal validation cohorts. Supplementary Figure S3 also shows the precision-recall curves for MGP–RNN versus the clinical scores and machine learning methods on the internal cohort.

**Figure 3.**
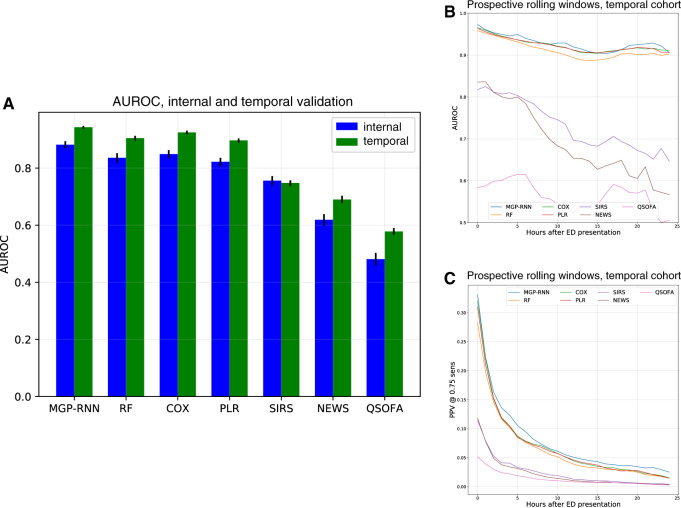
(A) Compares the AUROC obtained from the internal validation cohort with the AUROC from the temporal validation cohort for each method, along with bootstrap confidence intervals. (B) The AUROC as a function of hours after presentation to the ED for the temporal validation cohort for each method, limited to the first 24 h following initial presentation is shown. (C) The PPV at 75% sensitivity for each method as a function of number of hours after presentation to the ED is shown.

**Figure 4.**
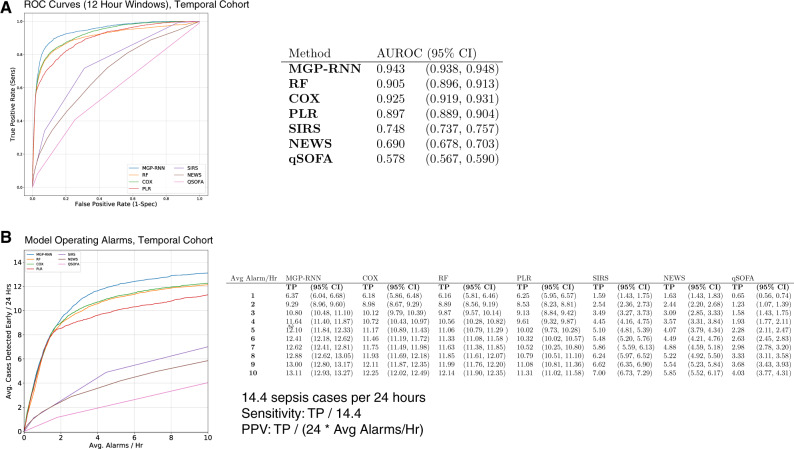
Results for the temporal validation cohort (analogous to Figures 1 and 2, which show results on the internal validation cohort.) (A) ROC curves and (B) the operating alarms are shown. There were an average of 14.4 sepsis cases per 24-h period in the dataset, so sensitivity can be recovered by dividing the reported *y*-axis value in panel B by 14.4. Positive predictive value at a particular threshold can be recovered by dividing the reported *y*-axis value by 24 times the reported *x*-axis value (ie, the average number of alarms per 24-h period).

When applied to the temporal validation cohort, MGP–RNN continues to outperform all 7 clinical risk score and machine learning comparisons. Figure 3A highlights the AUC for each approach across internal and temporal validation cohorts; discrimination generally improves on the temporal cohort. Figure 3B and C shows AUC and PPV as a function of hours after presentation to the ED. Not only does MGP–RNN discriminate better than all comparisons on a cohort of all comers to an adult ED, but MGP–RNN performs best across metrics at almost all points during encounters. Figure 4A and B illustrate the superior performance of MGP–RNN on a temporally distinct time period. Supplementary Figure S4 also shows the precision-recall curves for MGP–RNN versus the clinical scores and machine learning methods on the temporal cohort.

Additional results in the Supplementary Material show model interpretability, calibration, and the effect of shortening the size of the independent 12-h time windows used for evaluation ( Supplementary Figures S5–S7).

## DISCUSSION

We developed a deep learning approach to detect sepsis early and validated the model on a cohort of inpatient admissions as well as a temporal cohort of adults presenting to the ED. This approach uses comprehensive data from a patient’s hospital encounter to accurately detect sepsis from presentation to the ED until ICU transfer or hospital discharge.

Consistent with prior studies^16^^,^^32^^,^^35^ we find that machine learning models predict sepsis more accurately than clinical scores. These findings are clinically important, because qSOFA has been recommended as the screening tool for clinicians to use to identify patients for evaluation and potential escalation of care.^36^ We find that across metrics, qSOFA performs poorly at detecting sepsis early, also consistent with prior results.^37^ Health systems with fixed workforce capacity looking to implement clinical decision support within an EHR may consider investment in infrastructure to leverage machine learning methods. Otherwise, fixing the number of alerts per hour, we find that SIRS consistently outperforms qSOFA in detecting sepsis early.

Compared to previously published machine learning methods (CR, LR, and RF), we demonstrated the superior performance of MGP–RNN. Across AUC and operational metrics, MGP–RNN surpassed these methods to detect sepsis within 4 h. MGP–RNN detects more sepsis cases than other machine learning models at every number of fixed alarms per hour (Figure 2B). This performance gain is likely due to the coupling of the MGP with the RNN to better impute continuous functions for all vital sign and lab data. If a lab value is missing, the MGP will use learned relationships from the other available continuous features to calculate a distribution of possible values for the specific patient.

This study compared multiple previously published machine learning methods head-to-head on the same dataset, because comparing models across studies is non-trivial. Prior studies use a variety of outcome definitions, cohort definitions, model inputs, and statistical methods. Most sepsis models were developed on cohorts of ICU patients^16^^,^^30^^,^^38^^,^^39^ and nearly all use the publicly available MIMIC dataset.^40^ Many models use sepsis ICD codes as the outcome definition^38^^,^^39^^,^^41^^,^^42^ and predict sepsis at any point during an encounter, which is not directly actionable for frontline clinicians trying to follow SEP-1 bundle recommendations. In addition, nearly all models use static model inputs.^30^^,^^38^^,^^39^^,^^41–43^ While neural networks have been applied to sepsis prediction,^44^^,^^45^ none have been configured to use the entire time series of repeated measurements to detect sepsis within a window of time.

We further validated MGP–RNN on a more recent cohort that not only differs temporally but includes ED visits that do not result in admission. In comparison to the internal validation subset, performance characteristics improve for the temporal validation cohort. We suspect the improvement occurs because sepsis occurred in 19.0% of admitted patients, but only 6.4% of patients presenting to the ED. By including many low-risk patients, the improvement in AUC can be expected. The temporal validation results demonstrate the robustness of MGP–RNN within the implementation setting, where at the time of presentation it is unknown whether a patient will be admitted. The results further demonstrate MGP–RNN’s ability to detect sepsis better than all other methods at various points during the hospital course. These findings laid the groundwork for implementing MGP–RNN in the ED and a prospective evaluation is currently underway (ClinicalTrials.gov identifier: NCT03655626). Furthermore, our general approach can be scaled to other institutions, although each new local context would likely require retraining and possibly even the development of new models.

This study has a number of limitations. First, sepsis does not have a universally accepted definition. We adapted a definition similar to the clinical criteria outlined by CMS and this approach has potential weaknesses. Our definition does not address elevated but stable vital signs or abnormal laboratory values due to chronic organ dysfunction. We also did not include markers of acute respiratory dysfunction, a component of the CMS SEP-1 measure, due to variable reliability of data capture within our EHR. Although multiple sepsis definitions were compared in a prior analysis,^15^ a single definition was selected to train all machine learning models. Future work will have to assess model performance across multiple sepsis definitions. Second, this is a single-site study that describes development, internal, and temporal validation all within the same hospital. Another limitation of our study is the low PPV at high sensitivities; however, the low PPV is similar to other EHR-based sepsis prediction models.^46–48^

Although the model is not tested on a geographically distinct population, use of a temporal split cohort does demonstrate robustness of model performance.^49^ Future work with external partners to evaluate model performance will need to be conducted to demonstrate geographic generalizability. Furthermore, for models intended to be implemented within a local setting, we have previously shown that machine learning methods developed on locally curated EHR data can outperform models developed on national datasets.^19^ Finally, because MGP–RNN does not infer causal relationships, frontline clinicians will not have insight into factors driving sepsis risk. We do provide a variable importance graph in the Supplementary Figure S5, but the relationship between variables and sepsis is not necessarily causal.

In conclusion, this study couples probabilistic continuous function imputation for dynamic variables with a downstream deep learning model to calculate risk of sepsis. MGP–RNN is comprehensive, including repeated measurements of labs and vitals, as well as all administrations of medications from the entirety of a patient’s hospital encounter. We demonstrate that using our data elements and feature set, our modeling approach outperformed both clinical scores and previously published machine learning methods to detect sepsis early within cohorts of admitted patients and patients presenting to the ED.

## FUNDING

This work was partially funded by the Duke Institute for Health Innovation; HHS | NIH | National Institute of Allergy and Infectious Diseases (NIAID) grant number T32-AI007392 (to MEC); National Defense Science and Engineering Graduate (NDSEG) Fellowship (to JF); Duke Institute for Health Innovation Clinical Research & Innovation Scholarship (to NB, AL, KC, and MGS); and NSF Faculty Early Career Development Program (CAREER) Award (to KH).

## AUTHOR CONTRIBUTIONS

The authors meet criteria for authorship as recommended by the International Committee of Medical Journal Editors. ADB, JF, MEC, KC, MGS, and MS wrote the initial manuscript. JF designed and implemented the deep learning model and other machine learning models, conducted the evaluations, and did the statistical analyses. JF, MS, SB, MG, and MN designed the evaluation framework and experiments. KC, AL, MS, MG, MN, SB, MGS, and NB did the data preparation and data cleaning. KC and MS curated the temporal validation cohort. ADB, MEC, and CO’B did the manual clinical validation of the features. All authors contributed to the overall design of the study and contributed to the production of the final manuscript.

## SUPPLEMENTARY MATERIAL

Supplementary material is available at *Journal of the American Medical Informatics Association* online.

## CONFLICT OF INTEREST STATEMENT

ADB, JF, MEC, NB, AL, MG, MN, SB, KH, MS, and CO’B are the named inventors of the Sepsis Watch deep learning model, which was licensed from Duke University by Cohere Med, Inc. (but does not hold any equity in Cohere Med, Inc.). KC and MGS declared no conflict of interest statement. 

## Supplementary Material

ooaa006_Supplementary_Data
