# Supplementary material for: Machine learning for early detection of sepsis: an internal and temporal validation study
Source: JAMIA Open. 2020 Apr 11;3(2):252–60. doi: 10.1093/jamiaopen/ooaa006 (PMC7382639; doi:10.1093/jamiaopen/ooaa006)
Supplement: ooaa006_Supplementary_Data [file ooaa006_supplementary_data.zip › ooaa006-Suppl_Data/Supplemental Table 3.docx]

| **Definition** |  | **CDC Adult Sepsis Event** | | **Combo Values (95% CI)** |
| --- | --- | --- | --- | --- |
|  |  | Positive | Negative |  |
| **Sepsis-1** | Positive | 2,140 | 10,173 | Sensitivity: 0.647 (0.630-0.663)  Specificity: 0.744 (0.740-0.748)  PPV: 0.174 (0.167-0.181)  NPV: 0.962 (0.960-0.964) |
|  | Negative | 1,168 | 29,565 |  |
| **Sepsis-3** | Positive | 1,236 | 4,672 | Sensitivity: 0.374 (0.357-0.390)  Specificity: 0.882 (0.879-0.886)  PPV: 0.209 (0.199-0.220)  NPV: 0.944 (0.942-0.947) |
|  | Negative | 2,072 | 35,066 |  |
| **Duke Adult Sepsis** | Positive | 1,938 | 6,033 | Sensitivity: 0.586 (0.569-0.603)  Specificity: 0.848 (0.845-0.852)  PPV: 0.243 (0.234-0.253)  NPV: 0.961 (0.959-0.963) |
|  | Negative | 1,370 | 33,705 |  |
