# Supplementary material for: Machine learning for early detection of sepsis: an internal and temporal validation study
Source: JAMIA Open. 2020 Apr 11;3(2):252–60. doi: 10.1093/jamiaopen/ooaa006 (PMC7382639; doi:10.1093/jamiaopen/ooaa006)
Supplement: ooaa006_Supplementary_Data [file ooaa006_supplementary_data.zip › ooaa006-Suppl_Data/Supplemental Table 1.docx]

| **Criteria** | **Components of Criteria** |
| --- | --- |
| 2 or more SIRS criteria | Temperature >38C or <36C (6 hours)  Pulse >90 (6 hours)  Respiratory Rate >20 (6 hours)  WBC count >12 x 10^9/L, <4 x 10^9/L; or %bandemia >10% (24 hours) |
| Suspicion for infection | Blood culture order (24 hours) |
| 1 element of end organ failure | Serum creatinine >2.0 mg/dL (24 hours)  INR >1.5 (24 hours)  Total bilirubin >2.0 mg/dL (24 hours)  SBP <90 or decrease in SBP>40 mm Hg (6 hours)  Platelets <100 x 10^9/L (24 hours)  Lactate >2.0 mmol/L (24 hours) |
