# Supplementary material for: Machine learning for early detection of sepsis: an internal and temporal validation study
Source: JAMIA Open. 2020 Apr 11;3(2):252–60. doi: 10.1093/jamiaopen/ooaa006 (PMC7382639; doi:10.1093/jamiaopen/ooaa006)
Supplement: ooaa006_Supplementary_Data [file ooaa006_supplementary_data.zip › ooaa006-Suppl_Data/Supplemental Table 2.docx]

| **Variable Type (n)** | **Variables** |
| --- | --- |
| Demographics (3) | Age, Gender, Race |
| Other Encounter Info (4) | Admission Source, Admission Type, Weight at admission, Prior # Sepsis Encounters |
| Comorbidities (29) | Congestive Heart Failure, Valvular Disease, Pulmonary circulation disorders, Peripheral vascular disease, Hypertension, Paralysis, Other neurological disorders, Pulmonary circulation disorders, Diabetes without chronic complications, Diabetes with chronic complications, Hypothyroidism, Renal failure, Liver disease, Chronic peptic ulcer disease, HIV and AIDS, Lymphoma, Metastatic cancer, Solid tumor without metastasis, Rheumatoid arthritis / collagen vascular diseases, Coagulation deficiency, Obesity, Weight loss, Fluid and electrolyte disorders, Blood loss anemia, Deficiency anemias, Alcohol abuse, Drug abuse, Psychoses, Depression |
| Laboratory Values (29) | Albumin, ALT, Ammonia, AST, Bandemia, Bicarbonate, Bilirubin, BUN, CK-MB, Creatine Kinase, CRP, D-Dimer, ESR, Fibrinogen, Glucose, Hematocrit, INR, Lactate, LDH, Magnesium, PCO2, pH, Platelets, PO2, Potassium, Serum Creatinine, Sodium, Troponin, WBC |
| Continuous-valued Vital Signs (7) | Systolic BP, Diastolic BP, MAP, Pulse, Pulse Oximetry, Respiratory Rate, Temperature |
| Categorical-valued Vital Signs (2) | AVPU [alert, voice, pain, unresponsive] Score (binary: alert / other),  Any Supplemental Oxygen (binary) |
| Medications (10) | Antibiotics, Benzodiazepines, Chemotherapy, Heparins, Immunosuppressants, Insulins, IV Fluids, Opioids, Steroids, Vasopressors |
| Blood Culture Order Time (1) | Blood Culture (indicating suspected infection) |
| Missing Data Indicators (36) | Indicator variables for the 29 Laboratory Values and 7 Continuous-valued Vital Signs, indicating whether variable was measured in the last hour |
