# Supplementary figures and images for: Machine learning for early detection of sepsis: an internal and temporal validation study
Source: JAMIA Open. 2020 Apr 11;3(2):252–60. doi: 10.1093/jamiaopen/ooaa006 (PMC7382639; doi:10.1093/jamiaopen/ooaa006)

A

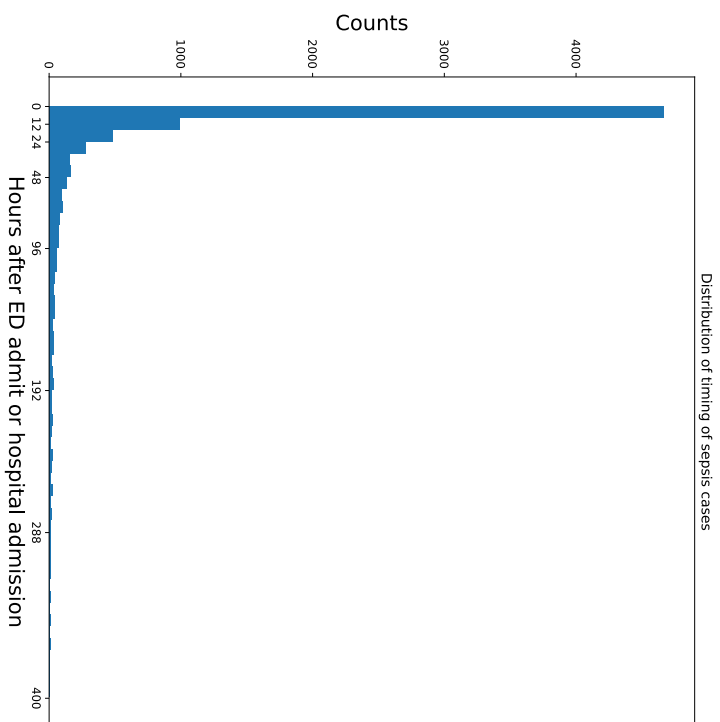

B

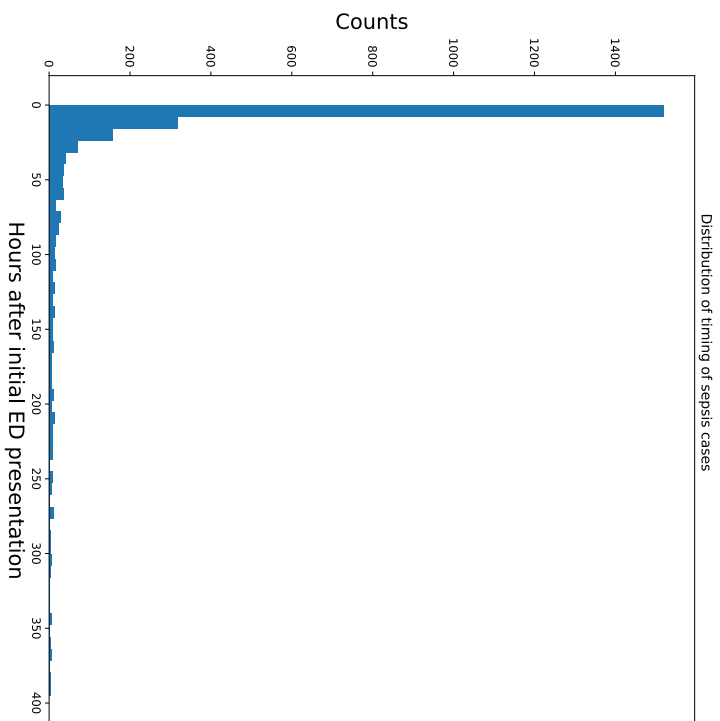

Supplement: ooaa006_Supplementary_Data [file ooaa006_supplementary_data.zip › ooaa006-Suppl_Data/Supplemental Figure 1.pdf]

A

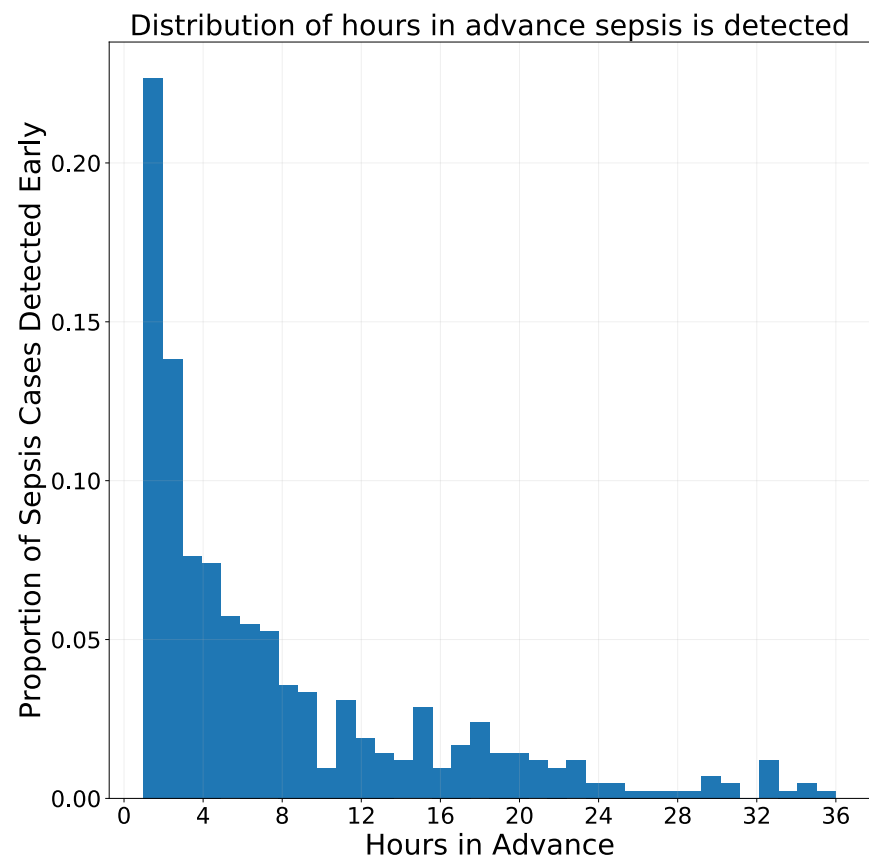

B

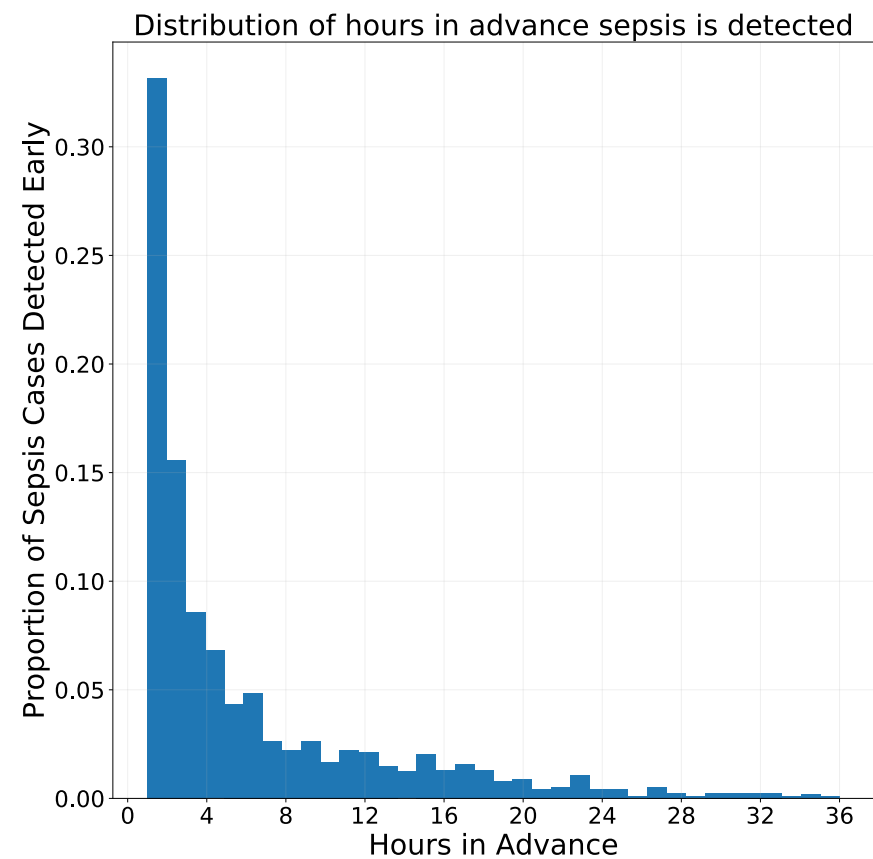

Supplement: ooaa006_Supplementary_Data [file ooaa006_supplementary_data.zip › ooaa006-Suppl_Data/Supplemental Figure 2.pdf]

# Precision Recall Curves (AUPR), Internal Cohort

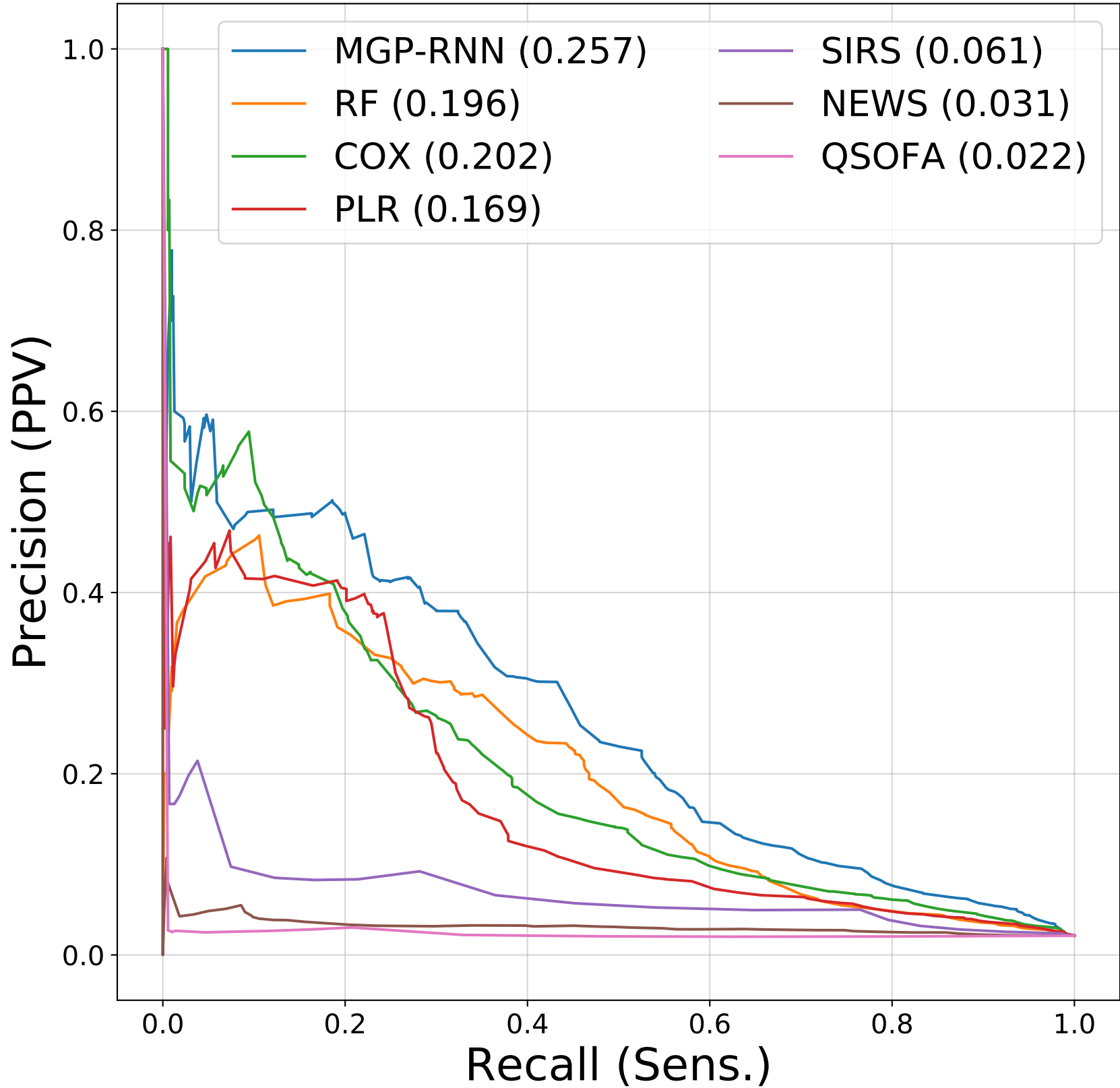

Supplement: ooaa006_Supplementary_Data [file ooaa006_supplementary_data.zip › ooaa006-Suppl_Data/Supplemental Figure 3.pdf]

# Precision Recall Curves (AUPR), Temporal Cohort

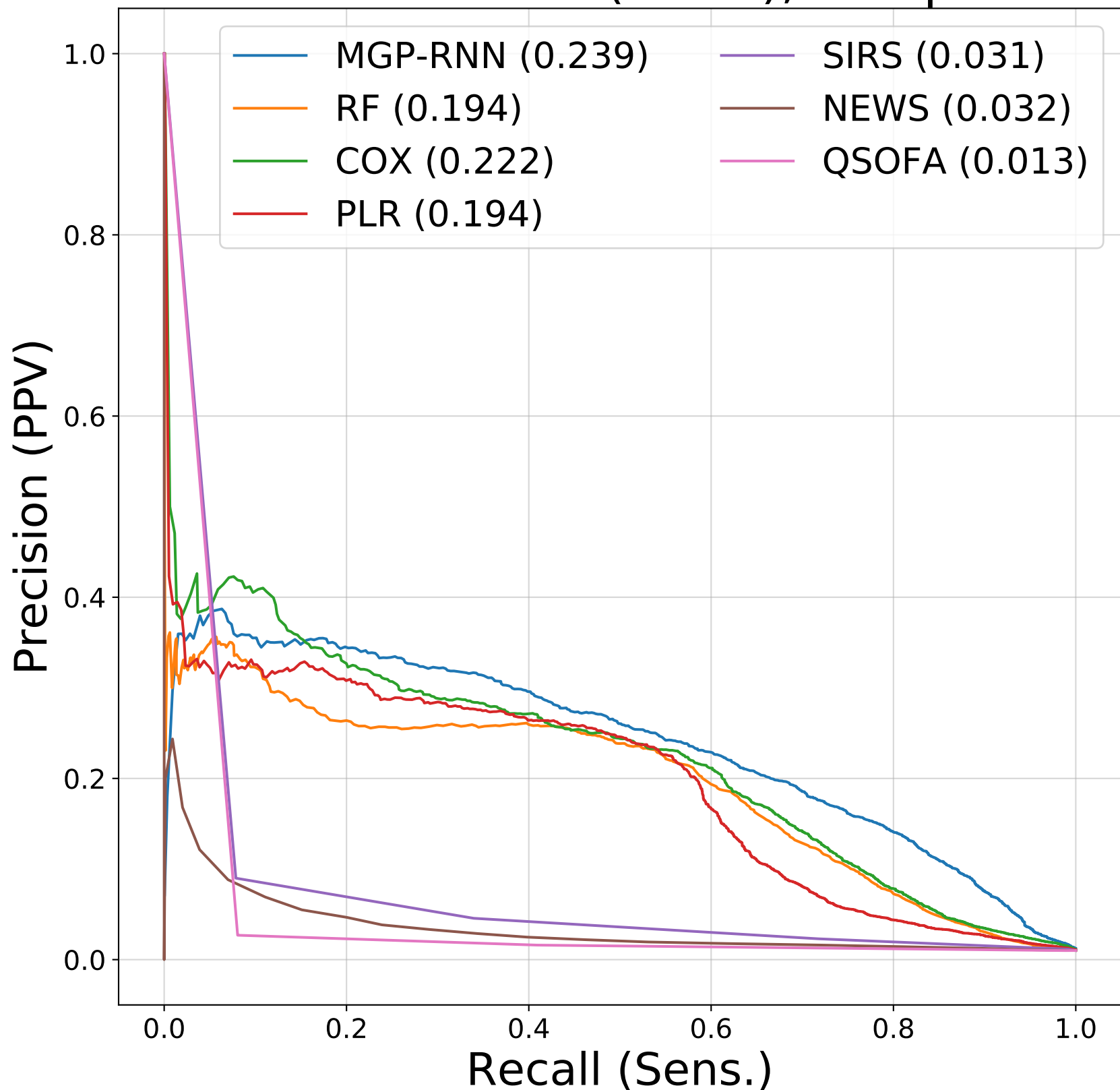

Supplement: ooaa006_Supplementary_Data [file ooaa006_supplementary_data.zip › ooaa006-Suppl_Data/Supplemental Figure 4.pdf]

# Variable importances from ranks of input gradients

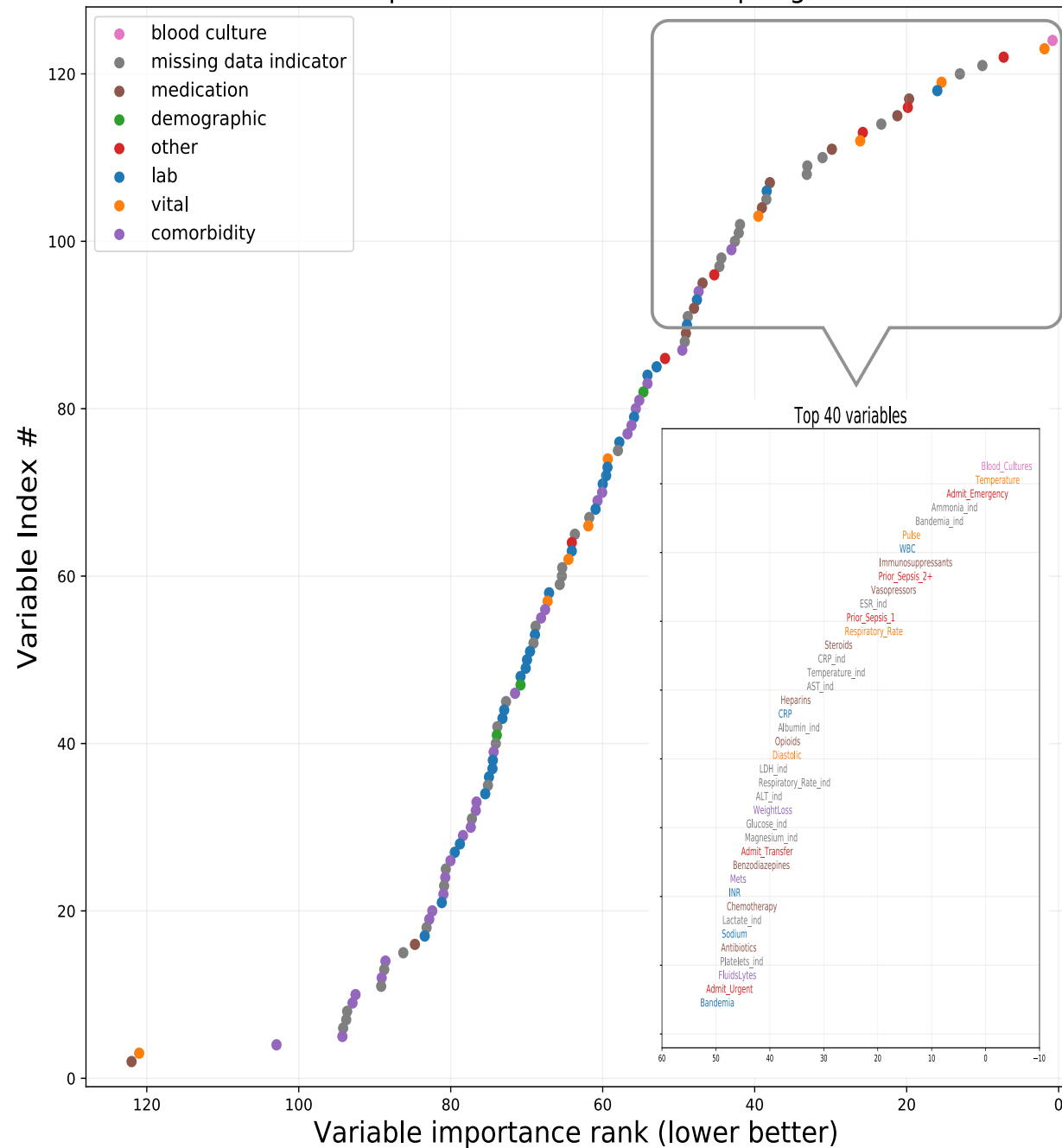

Supplement: ooaa006_Supplementary_Data [file ooaa006_supplementary_data.zip › ooaa006-Suppl_Data/Supplemental Figure 5.pdf]

A

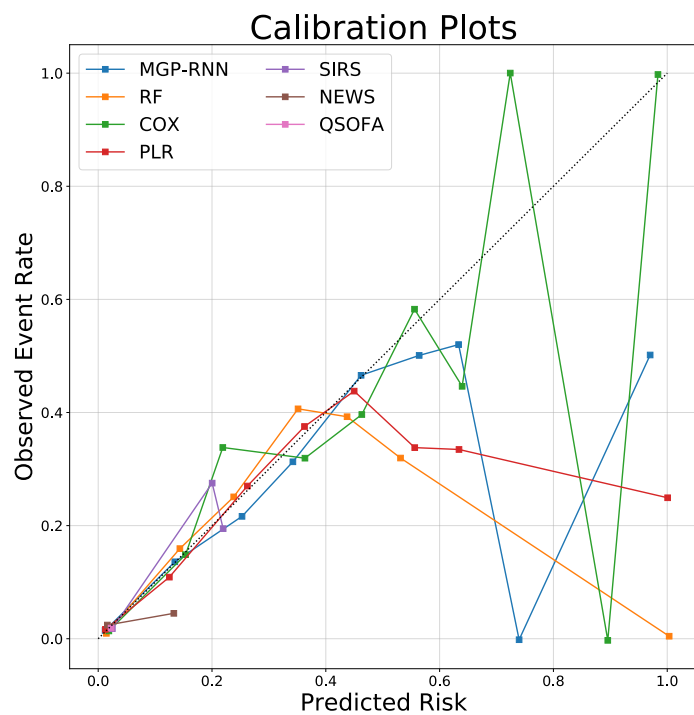

B

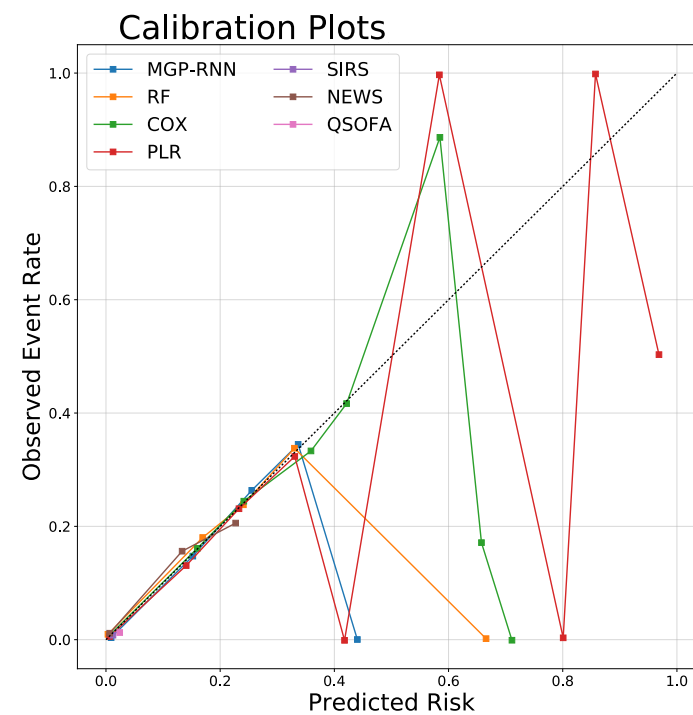

Supplement: ooaa006_Supplementary_Data [file ooaa006_supplementary_data.zip › ooaa006-Suppl_Data/Supplemental Figure 6.pdf]

# Varying the Evaluation Window Size

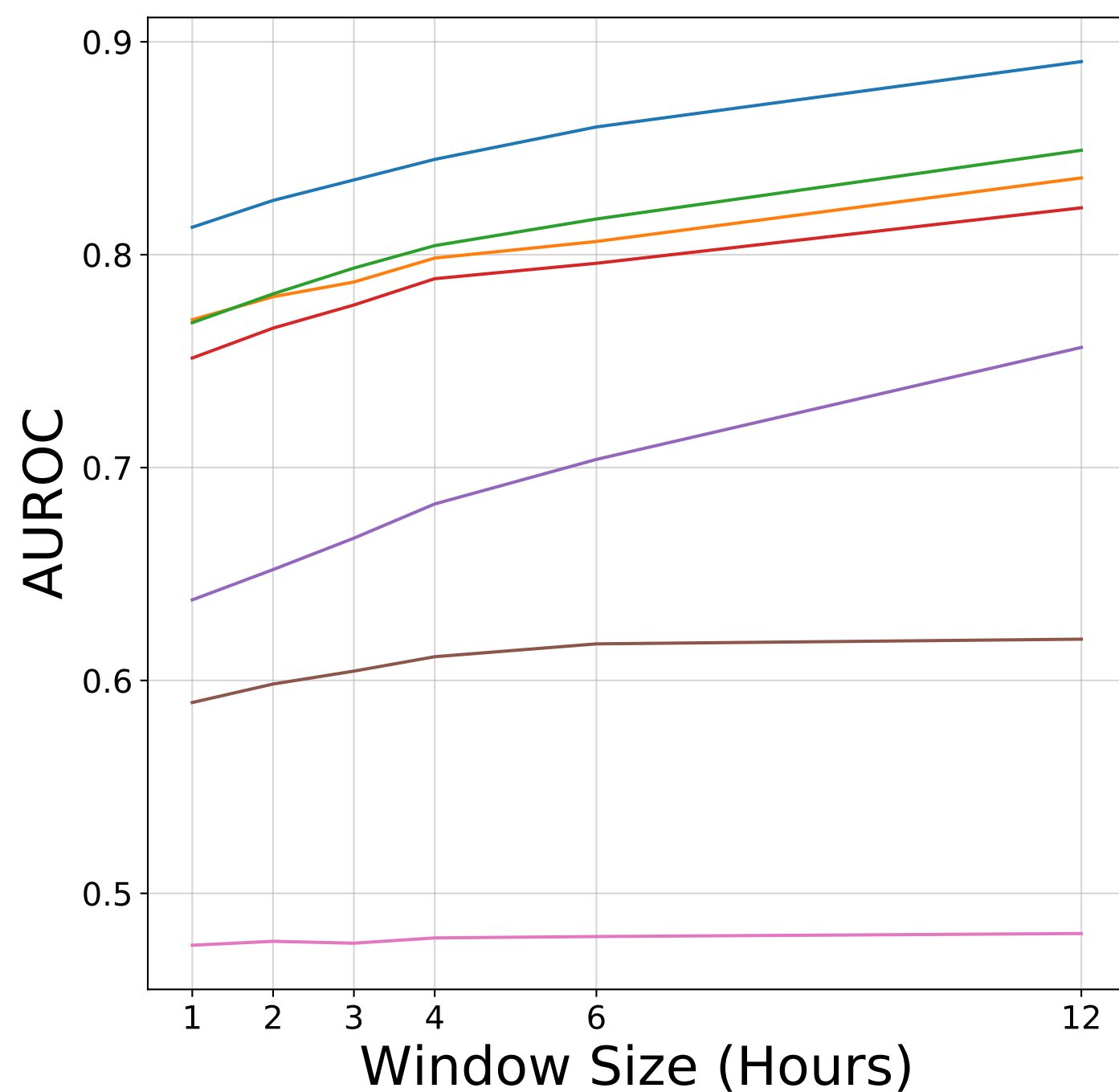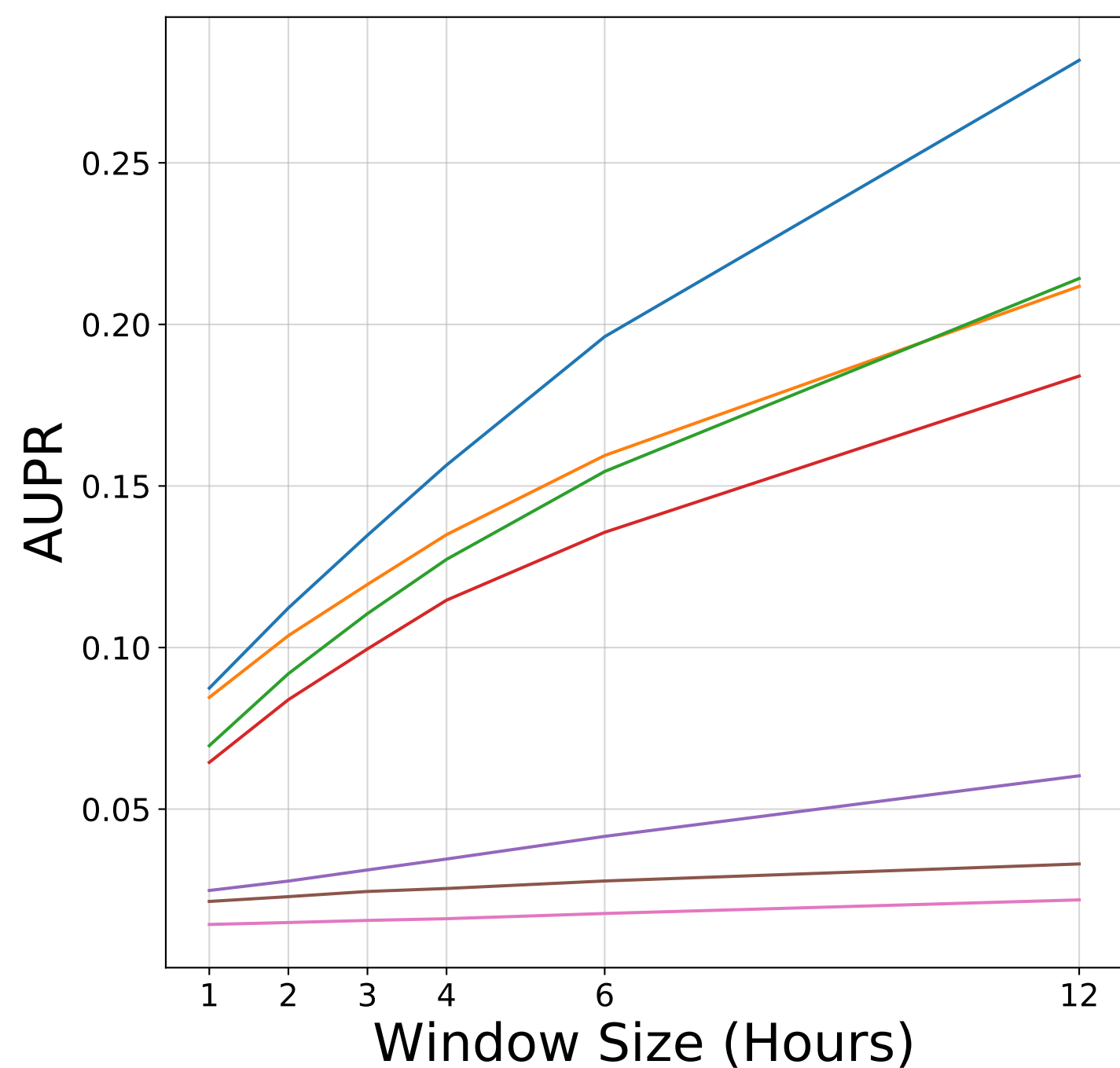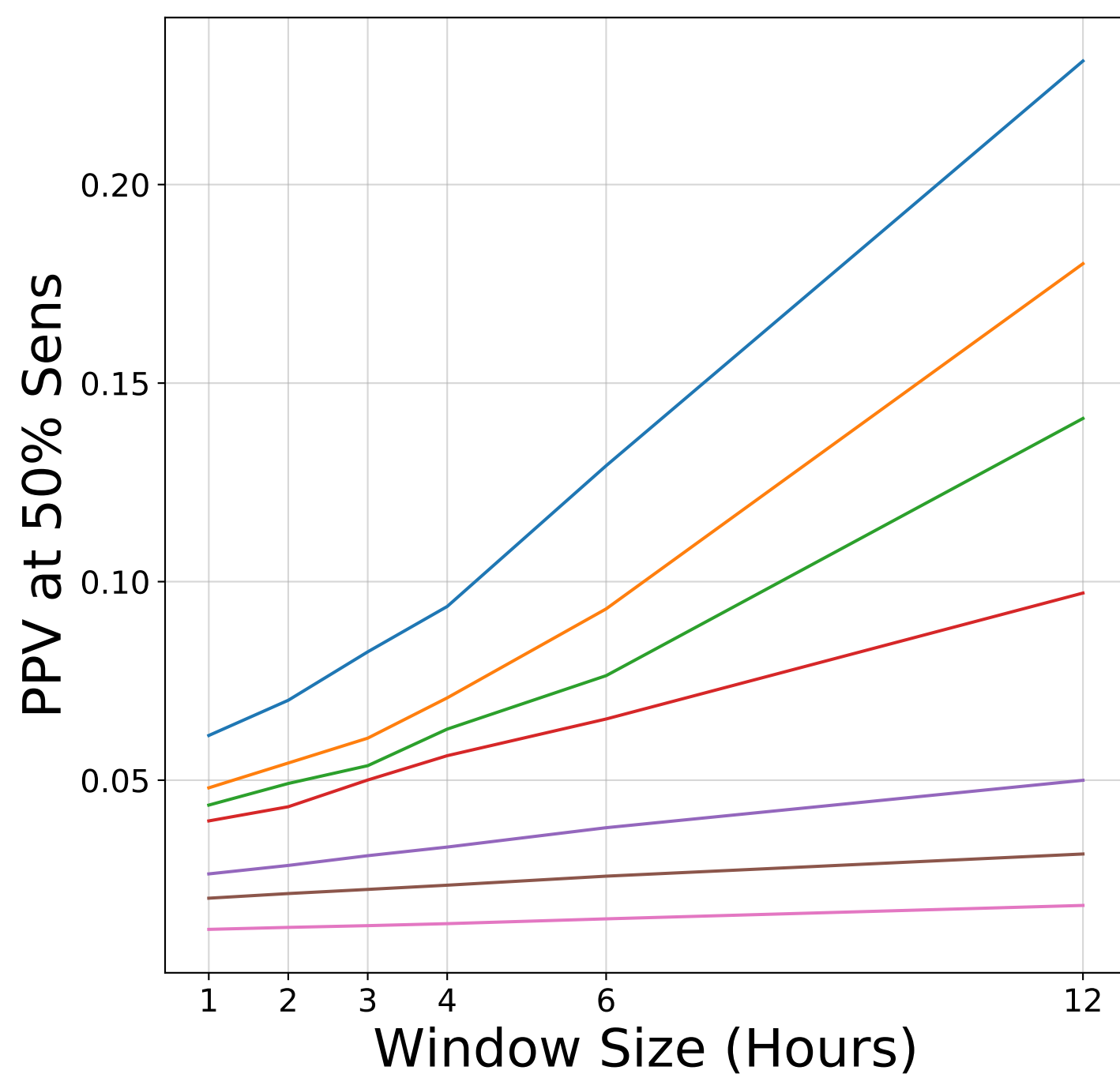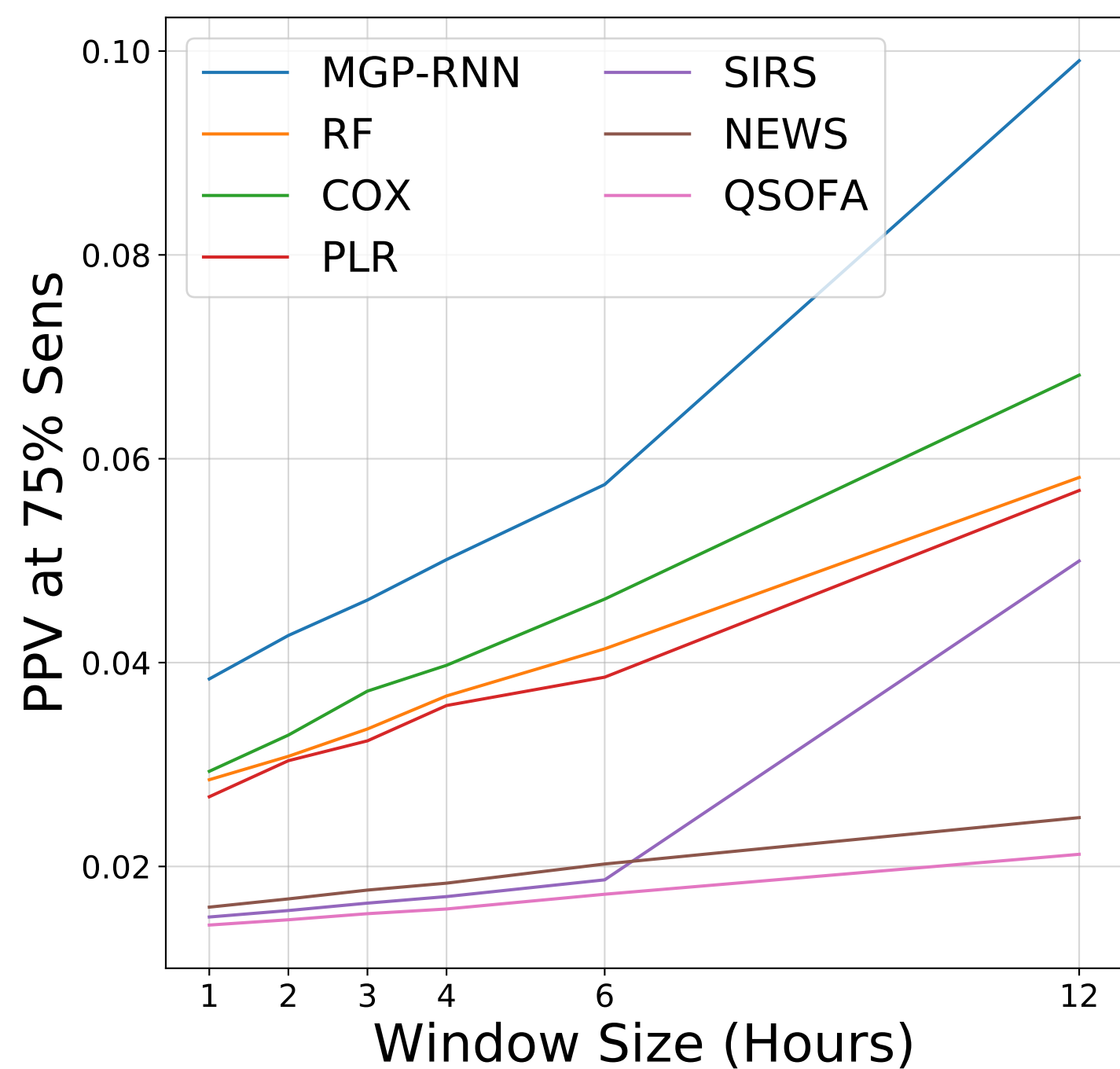

Supplement: ooaa006_Supplementary_Data [file ooaa006_supplementary_data.zip › ooaa006-Suppl_Data/Supplemental Figure 7.pdf]
